# Drug Resistance in Nontuberculous Mycobacteria: Mechanisms and Models

**DOI:** 10.3390/biology10020096

**Published:** 2021-01-29

**Authors:** Saloni Saxena, Herman P. Spaink, Gabriel Forn-Cuní

**Affiliations:** Institute of Biology Leiden, Animal Science and Health, Leiden University, Einsteinweg 55, 2333CC Leiden, The Netherlands; s.saxena@umail.leidenuniv.nl (S.S.); h.p.spaink@biology.leidenuniv.nl (H.P.S.)

**Keywords:** nontuberculous mycobacteria, drug resistance mechanisms, antimicrobial testing, drug discovery

## Abstract

**Simple Summary:**

Recently, there has been a considerable rise in infections caused by nontuberculous mycobacteria (NTM). These mycobacteria, which comprise a large and diverse range of species, have developed resistance to most conventional antibiotics, rendering their treatments unsatisfactory. This review summarizes the mechanisms and strategies adopted by NTMs to evade the action of antimicrobial drugs and techniques that can be used to develop better therapies against them. We also suggest some ways to accelerate the drug development pipeline by utilizing a combination of computational, laboratory and animal testing methods.

**Abstract:**

The genus *Mycobacteria* comprises a multitude of species known to cause serious disease in humans, including *Mycobacterium tuberculosis* and *M. leprae*, the responsible agents for tuberculosis and leprosy, respectively. In addition, there is a worldwide spike in the number of infections caused by a mixed group of species such as the *M. avium*, *M. abscessus* and *M. ulcerans* complexes, collectively called nontuberculous mycobacteria (NTMs). The situation is forecasted to worsen because, like tuberculosis, NTMs either naturally possess or are developing high resistance against conventional antibiotics. It is, therefore, important to implement and develop models that allow us to effectively examine the fundamental questions of NTM virulence, as well as to apply them for the discovery of new and improved therapies. This literature review will focus on the known molecular mechanisms behind drug resistance in NTM and the current models that may be used to test new effective antimicrobial therapies.

## 1. The Rise of Nontuberculous Mycobacteria

Mycobacteria are a large group of non-motile, rod-shaped bacteria that tend to grow mold-like pellicles on liquid culture media. Out of the 150 species known to this genus, nearly 25 are known to cause disease in humans. The most well-known mycobacteria species are the *M. tuberculosis* and *M. leprae* complexes, with an estimated prevalence rate of 130 (the year 2020) and 2 (the year 2018) cases per 100,000 population, respectively [1,2], while all others are collectively called nontuberculous mycobacteria (NTMs) [3]. Despite that NTMs are less widespread pathogens for humans than *M. tuberculosis*, they have proven to be an emerging threat to the immunocompromised population [4], with an estimated 4.1–14.1 cases per 100,000 population worldwide (2013) [5]. NTMs are ubiquitous and can survive in a wide range of environmental conditions, and their infections are difficult to diagnose [6]. The most common NTM-related pathologies are pulmonary infections (pulmonary nontuberculous mycobacterial disease) caused by strains from the *M. avium* complex and *M. abscessus* [6,7], but NTMs can also cause skin and soft tissue infections (e.g., *M. marinum* infection and Buruli ulcer caused by *M. ulcerans*), lymphadenitis in immunocompromised children, and even invasive disseminated disease eventually leading to death.

According to Runyon, NTMs can be classified based on the growth rate and pigment formation (Table 1) [8]. Types I, II, and III strains are classified as slow-growers because they take seven or more days of growth for forming visible colonies on a subculture plate [9]. They are differentiated on their ability to produce pigments only on exposure to light (type I or photochromogens) or also in the dark (type II or scotochromogens), or not being strongly pigmented (type III or non-photochromogens) [10]. Type IV strains are regarded as rapid-growers as they take less than seven days to form visible colonies on a subculture plate [10]. Generally, slow-growing mycobacteria are much more prevalent than fast-growing ones [11] and present higher ratios of drug resistance (with the fast-growing *M. abscessus* being a notable exception) [12]. It has been suggested that all mycobacteria evolved from a common ancestral rapid growing mycobacterial strain [13,14,15].

Recently, there has been a considerable increase in the number of reported NTM related diseases, including respiratory infections caused by various strains from the *M. avium* complex, *M. kansasii* and *M. abscessus* [25]. This is partly because of the awareness of the symptoms caused by these infections and improvements in detection techniques, but also because of an increase in the number of susceptible individuals and that NTM can form biofilms in common household and hospital sources of infection (such as showerheads, faucets, water distribution systems, plumbing systems, etc.) [26,27]. The situation is worrying because, just like tuberculosis, these bacteria have developed high resistance against conventional antibiotics [28]. However, these pathogens are still considered opportunistic since they require a combination of constant exposure as well as host susceptibility to infection, and these infections have mainly remained limited to patients with pre-existing lung diseases [25,29].

The major NTM that is infecting such individuals suffering from chronic diseases like cystic fibrosis is *M. abscessus*, which is a rapidly growing, intrinsically multidrug-resistant species [30]. These infections are often impossible to treat despite prolonged antibiotic therapy, and the therapy may even be contraindicated with lung transplantation, leaving no effective options for treatment [31]. While NTM infections were earlier thought to be independently acquired by susceptible individuals, the recent consensus is that such infections are frequently transmitted indirectly from an infected to a healthy individual, for instance, via contaminated hospital equipment [32]. Some opportunistic infectious NTM species tend to cluster in specific geographical distributions, and there may be a genetic basis for the susceptibility to their infection in particular patients [11,33,34]. Finally, relapse and reinfection is a major problem with some NTM infections, like the ones caused by *M. avium* complex [35], although it is less so for other species like *M. kansasii* [36].

Currently, the treatment for almost all NTM infections is based on macrolide-based antibiotics, such as clarithromycin or azithromycin. For NTM infections caused by the slow-growing group, the regime also includes ethambutol and rifampicin [37], while for fast-growers, it includes an aminoglycoside and either cefoxitin, imipenem or tigecycline [38]. These treatments are largely empirical, derived from years of clinical practice, can last for as long as 18 months, are costly, and are often associated with drug-related toxicities and side-effects [39]. Cure rates range from 80–90% with *M. malmoense* infections to just 30–50% with *M. abscessus* infections [40]. Thus, the discovery of new and more efficient therapies against NTMs is an important topic of research. However, a major bottleneck is the low susceptibility of mycobacteria to most antibiotics, including the ones used against tuberculosis [41]. A better understanding of the underlying mechanisms behind this drug resistance by improving the available models to study their infection could significantly help in accelerating the drug discovery process.

## 2. Mechanisms of Drug Resistance in Nontuberculous Mycobacteria

Drug Resistance can be either intrinsic (natural) or acquired [42]. Intrinsic resistance describes a situation where an organism possesses a set of special features that allows it to tolerate a particular drug or survive in an otherwise hostile chemical environment [42]. Mechanisms by which NTMs are intrinsically resistant to antibiotics include their thick, impermeable cell walls or their presence in biofilms and granulomas, which effectively decrease drug uptake, as well as the expression of proteins that specifically target clinically used antibacterial compounds.

On the other hand, acquired resistance refers to the case where a resistant strain emerges from a population that was previously drug-sensitive [42]. These events are usually related to the prolonged antibiotic treatments required to cure NTM infections. The acquired resistance is particularly severe for NTMs that only have a single copy of genes encoding common target proteins such as ribosomes, thus increasing the risk of acquiring protective mutations with single-drug treatments [4,43]. Here, we will focus on the mechanisms of mycobacterial physiology that make them naturally resistant to antimicrobial treatments since Nasiri et al. recently reviewed the mutations that may cause resistance to certain antibiotics in NTM [44].

**Figure 1:**
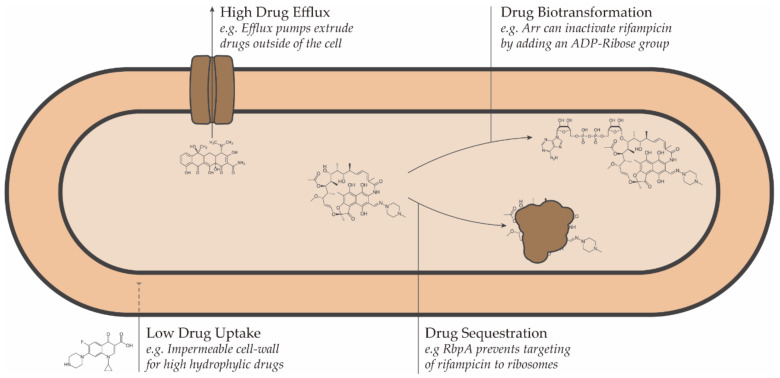
Schematic representation of the intrinsic drug resistance mechanisms in bacteria.

Conceptually, resistance to antimicrobial drugs can be a result of one or more of the following mechanisms: decreased drug uptake, increased drug efflux, increased drug metabolism, or reduced drug sequestration (Figure 1) [45].

### 2.1. Drug Uptake 

One of the most important factors responsible for the natural resistance of mycobacteria is its thick impermeable cell wall, which has an unusual structure: the peptidoglycan contains N-glycolyl muramic acid instead of the usual N-acetyl muramic acid, and the most abundant lipids are long-chain saturated fatty acids containing up to 90 carbons [46]. This causes an exceptionally high degree of hydrophobicity in comparison to the cell wall of other bacteria and therefore affects the uptake of compounds from the environment. For example, the rate of uptake of charged compounds in NTM can be as low as 1% of the rate of uptake in *E. coli* [46].

The outer membrane constitutes nearly a third of the total mycobacterial cell weight [47], and thus a great part of the energy generated by mycobacteria is used in cell wall synthesis and repair [48]. Consequently, mycobacteria with thick cell walls have less available energy for the production of new cells and thus show slower growth. This may help in explaining why slow-growing NTMs are generally more drug-resistant and persist more easily in a dormant state than their fast-growing counterparts [12,49]. The importance of the composition and permeability of the mycobacterial cell wall in its homeostasis is evidenced by the fact that the inactivation of genes traditionally related to metabolism also selectively affect the cell wall structure. For example, the inactivation of AsnB, which encodes an asparagine synthetase that is responsible for amino acid metabolism, disrupts the cell wall structure, thereby conferring hypersensitivity towards hydrophobic drugs in *M. smegmatis* [50]. Similarly, PknG plays an important role in imparting intrinsic resistance in *M. smegmatis* to multiple antibiotics by controlling the cell envelope structure, in addition to its role in cell metabolism [51].

In addition to proteins related to metabolism, the activity of proteins directly involved in the cell wall structure such as MtrAB [52,53], Pks12 and Maa2520 [54], and Fbpa [55] greatly affects the sensitivity of mycobacteria to hydrophilic drugs. Interestingly, deletion of the gene encoding Fbpa renders *M. smegmatis* particularly susceptible not only to hydrophilic antibiotics but also to hydrophobic ones because of the resulting increased fluidity of the envelope [56].

On the other hand, the thick mycobacterial cell wall not only provides a barrier for stressors but can also make it difficult for the bacilli to take up nutrients from the environment. Often, mycobacteria overcome this problem by the synthesis of porins–proteins that provide a narrow channel for the uptake of nutrients [57]. The expression of these porins in NTM has been linked to their growth rate [58]. Importantly, these porins provide a channel through which some antimicrobial compounds can enter into the mycobacterial cell [59]. For example, *M. smegmatis* mutants lacking porins have higher survival rates inside phagocytic cells, presumably by evading the inflow of antimicrobial peptides and lysosomal enzymes [57,60]. Likewise, these porins can be entry points for small hydrophilic drugs like norfloxacin, chloramphenicol and β-lactam antibiotics. The loss of specific porins in *M. smegmatis* reduces the permeability of hydrophobic drugs (like vancomycin, erythromycin and rifampicin [61]) and significantly decreases the bacterial sensitivity to these antibiotics without altering the activity of their targets, thus causing a significant rise in the resistance to these antibiotics [61,62].

In addition to the cell wall, two of the most characteristic mechanisms to promote antimicrobial resistance in NTMs are related to their colony behavior: the formation of biofilms and granulomas.

NTMs are efficient biofilm producers, as evidenced by their frequent recovery from surfaces of, for instance, water pipes, showerheads and healthcare equipment. [63,64,65]. Some studies indicate a link between biofilm formation and pathogenicity [66,67,68]. Biofilms enable NTMs to tolerate high dosages of antibiotics in their immediate environment: cells in biofilms are at least 10 times more tolerant than suspension-grown (planktonic) bacteria [69]. The precise reason behind this remains elusive, although it is speculated that the waxy lipid-rich extracellular matrix of the biofilm creates a strong physical barrier that blocks the penetration of drugs [41]. The potential increased horizontal gene exchanges between the closely interacting bacteria in the biofilms may also help with the spread of drug resistance [70]. Moreover, several species undergo actual cellular changes during biofilm formation that may be linked to the development of adaptive resistance, which is reversed when the bacteria are removed from the biofilm [71]. This may be due to the fact that some genes are expressed differently when the bacteria are grown in biofilms than in suspensions [72]. For instance, it has been suggested that the increased chlorine resistance of *M. avium* and *M. intracellulare* cells grown in biofilms is attributable to the changes in the cell wall, which in turn results from alterations in mycolic acid structures [73].

The formation of granulomas is the immunological hallmark of most mycobacterial infections. Essentially, a granuloma is a microenvironment comprising a variety of different immune cells that entrap the infecting bacilli to contain its spread [74]. Structurally, it mainly comprises macrophages, epithelioid cells and multinucleated giant cells, surrounded by a layer of T-lymphocytes [75,76]. Although NTM infections are more commonly associated with alveolar granulomas, disseminated NTM diseases sometimes result in granulomas in other parts of the body like the liver, especially in people with a history of tumors [77]. These structures present a major challenge to NTM drug therapy in two ways: they limit the penetration of the drugs into the bacteria inside the immune cells, and the anoxic conditions in the granuloma center promote physiological and morphological states that make them more tolerant [41]. Although granulomas can be viewed as a host defensive structure intended to eliminate the pathogen, they can also provide a niche for the prolonged survival of mycobacteria in the body: mycobacteria can survive for years in a latent state within the granuloma [78]. Eventually, the death of the infected cells in the granuloma creates a necrotic zone that disintegrates, thus providing an exit route for the latent bacteria to release back into the lung [79].

### 2.2. Drug Efflux

In addition to the cell wall’s restricting capacity for entry of potentially harmful molecules into the cell, mycobacteria utilize efflux pumps to remove unwanted molecules that may still get inside [44]. From a biological perspective, efflux pumps are essential for physiological processes like cell-to-cell communication, cellular homeostasis, detoxification of intracellular metabolites and intracellular signal trafficking [80]. However, they also extrude drugs from the periplasm to the outside of the cell, rendering them ineffective. As a consequence, deletion of specific efflux pumps in *M. smegmatis* increases its drug sensitivity by as much as two to eight times [81]. Many efflux pumps have limited substrate specificity and can expel a wide range of structurally dissimilar substrates, thus conferring resistance to multiple drugs at once [44]. This efflux-mediated resistance has been reported for a variety of drugs as fluoroquinolones [82], tetracyclines [83,84], erythromycin and rifamycins [81].

### 2.3. Drug Transformation and Sequestration

Enzymatic biotransformation of drugs into compounds having much lower antimicrobial activity on several mycobacterial species has been described for penicillin, fluoroquinolones, aminoglycosides, and rifampicin. Due to the presence of β-lactamase enzymes in mycobacteria, most β-lactam antibiotics such as penicillin and cephamycin cannot be used in the treatment of mycobacterial infections [85], although some exceptions like cefoxitin and imipenem with modified structures resistant to β-lactamase activity are still used [86,87]. Because of this reason, most β-lactams only show significant activities when used in combination with β-lactamase inhibitors [88,89].

An important class of deactivating enzymes include transferases that modify the drug in such a way that it becomes ineffective. Prominent among them are acetylating enzymes, which are responsible for imparting resistance in most mycobacterial species against a variety of drugs, including fluoroquinolones [90], isoniazid [91] and aminoglycosides [92,93]. Similarly, enzymes that modify drug compounds by transferring nitroso and phosphate residues have been identified for fluoroquinolones and aminoglycosides, respectively [90,93]. For NTMs like *M. smegmatis* and *M. abscessus*, the major determinant of innate resistance towards macrolide and rifampicin are Erm methyltransferase and ADP-ribosyltransferase (Arr), respectively [94,95,96]. The resistance to macrolides is particularly significant, given that most NTM therapies involve the use of macrolides as first-line drugs [97]. The *erm* genes cause methylation of the 23S ribosomal RNA, which in turn prevents the binding of macrolides to their target, the ribosomes. However, this is not the only mechanism that confers macrolide resistance. Mutations in the 23S ribosome itself often render macrolides ineffective [98,99]. These mutations are frequent in mycobacteria because they possess only one or two rRNA operons, and mutation in any one of them can sufficiently alter the ribosome in a way that the macrolide can no longer bind to it [100].

Although rifampicin remains a front-line drug for the treatments in most NTM infections, instances of acquired resistance in *M. avium* complex and *M. kansasii* are reported [44]. Mutations in the target of rifampicin (rpoB gene) are generally held responsible for this [101,102,103]. It also has been observed that the preference of rifampicin to inhibit one of the two rpoB promoters over the other facilitates increased rpoB expression from the latter, leading to the growth of more resistant lines [104]. However, recent studies suggest that some other mechanisms might be at play. It has been suggested that RNA polymerase binding protein A (RbpA) can shield the target from rifampicin by either overlapping with its binding site or causing a conformational change to prevent any interaction [105].

## 3. Models for Drug Discovery against NTM

There is a wide range of techniques that can be employed in the development of new potential antimicrobial therapies against NTMs. These techniques can be classified depending on the tools employ between in silico, in vitro, or in vivo. In general terms, in silico techniques are useful to generate new leads and narrow the search of potential candidates based on prior information at the start of a study or to optimize compounds based on specific targets via virtual simulations. These leads can then be tested for efficacy using standardized in vitro analysis, which allows the determination of their potential antimycobacterial activity. Finally, in vivo animal models can be used to recreate infection environments and are therefore interesting for preclinical evaluation of potential compounds. We summarized the main attributes for each category in Table 2. A recent review by Rampacci et al. explains in detail the different techniques, assays, and preclinical models against NTMs that have been developed, with an emphasis on the newer models [106]. We direct the reader to that review for an in-depth description of these methodologies and their read-outs. Here, we will give a brief outline of the most common techniques implemented in the lab and how they complement each other to create an integrated pipeline for drug discovery.

### 3.1. In Silico Predictions

Computational methods are commonly used in the drug discovery process for the identification of suitable drug targets. The targets can be identified at different levels, ranging from molecular to cellular to whole-organism levels [107]. Once these targets are identified, suitable molecules that interfere in its working can be identified [108]. In silico methods can facilitate faster drug development by making predictions for a large set of drug candidates without the need of chemically synthesizing each of the compounds [109]. Moreover, they can bring in-depth molecular level insights that can allow for even more targeted drug development [110].

These methods can be especially useful in the case of strains that are not culturable or have long cultivation periods. For instance, the tuberculous mycobacteria *M. leprae* has an extremely slow doubling time in almost all available growth media and can only be inoculated in cold-like environments like the body of armadillos or hind footpads of mice [111]. Recent studies have demonstrated the importance of computer simulations in understanding drug resistance in *M. leprae*. Using molecular docking simulations, it was shown that certain drugs like rifampicin and ofloxacin bind less effectively to the drug-resistant mutant *M. leprae* strain as compared to the native strain due to loss of a hydrogen-bonding site in the target of the drug [112,113,114].

This approach to drug discovery is generally divided into two categories depending on if the 3D structure of the target is known or not: structure-based drug discovery (SBDD) and ligand-based drug design (LBDD), respectively [115]. SBDD methods are useful to discover the molecular basis of drug action or to optimize compound derivatives for a specific species. Usually, the target 3D structure—identified either from experimental data such as nuclear magnetic resonance (NMR) or X-ray diffraction spectroscopy or through homology modeling—is used to identify potential binding pockets [116,117]. On a molecular level, these simulations can elucidate how a point mutation on a target can lead to structural variation, which ultimately influences the effectiveness of a drug [118]. This approach was successfully used to evaluate 11 tetrahydropyridine compounds as antimicrobials for *M. abscessus* [119]. Since the mechanism of action of THP is known–inhibiting the efflux pumps MmpL5 and Tap–, the binding sites for the drugs were identified on the pumps by docking simulations. Another method, molecular dynamics simulations [120], was recently employed to understand how the “predisposing” proteins present in certain populations make them susceptible to *M. avium* subsp. *paratuberculosis* infections [121]. The simulations could identify the exact residues where binding of mycobacterial and host proteins take place, which may open possibilities to target it specifically by suitable drugs.

Contrasting, LBDD methods can be employed even when the 3D structure of the target is not known, thus being excellent tools for the generation of initial leads. Essentially, from previously known ligand structures and their bioactivities, predictive models are created, which can be subsequently used to assess the viability of new ligands [122]. Most frequently, LBDD uses the structure–activity or structure–property relationship (SAR/SPR) studies, wherein the chemical structure of the ligand is correlated to its activity (or property) from a model developed from previously acquired data. The SAR approach was successfully used to evaluate a series of piperidinol derivatives [123], based on a previous finding that piperidinol efficiently works against *M. abscessus* and *M. tuberculosis* by targeting the mycolic acid transporter MmpL3 [124]. A series of similar compounds were synthesized and tested in vitro, and the data were used to create a SAR model to guide the design of subsequent derivatives, as well as to identify the molecular sites that can be effectively modulated.

Finally, comparative genetics is an important tool that should be explored further. In this technique, the genomes of pathogenic species are compared with non-pathogenic species to identify unique genes that encode potential virulence factors [125]. For example, a study of the genome of *M. abscessus* has led to the identification of several “non-mycobacterial” virulence genes that are likely acquired by the horizontal gene transfer (HGT) from other pathogens like *Pseudomonas aeruginosa* or *Burkholderia cenocepacia* [93]. These virulence factors can be an important target for potential new drugs and vaccines [126]. An important challenge in the discovery of new drugs for NTM is the lack of whole-genome information on different strains, although the situation is rapidly changing [127]. Indeed, this can mark a paradigm shift in NTM drug discovery as WGS has been shown to predict species and drug susceptibility with remarkably high accuracy [128,129].

### 3.2. In Vitro Susceptibility Testing

The first step towards the prediction of success or failure of a new antibiotic therapy is antimicrobial susceptibility testing (AST) in vitro. These tests measure the growth response of isolated organisms to a particular antimicrobial therapy. They are relatively cheap, easy to replicate, and scalable [130]. In addition, they are also relatively fast: the optimal incubation times for the broth microdilution method range from 7 days (at 28 to 30 °C for *M. marinum*) but can reach 6 weeks for the slower growers like *M. ulcerans*. The most widely accepted protocol for AST of NTMs is published by the Clinical and Laboratory Standards Institute (CLSI), which has recommended the use of microdilution as the gold standard for the determination of antibacterial susceptibilities [131]. Other methods are not recommended for testing antimicrobial effects against NTMs. For example, although commonly used for *M. tuberculosis*, the proportion method often yields misleading results for NTMs [132]; the agar disk diffusion method carries the inherent difficulty in the interpretation of zones of inhibition, especially when the amount of drug in the disk is near the breakpoint of the drug [133]; and the epsilometer test is quite rapid and simple, suffers from lack of reproducibility and exaggeration of drug susceptibility as determined by other techniques due to tailing of the ellipses [134,135].

There is controversy about the role of in vitro susceptibility testing for NTM diseases. This is mainly due to the unpredictable correlation between in vitro and clinical outcomes: correlation is particularly poor for *M. abscessus* and *M. simiae*, while it is reasonably satisfactory for *M. kansasii*, *M. marinum* and *M. fortuitum*, and for other species such as *M. avium* complex, the correlation holds good only for certain drugs like macrolides, but not for others [4]. This disconnect probably stems from a multifactorial origin ranging from strain selection and testing conditions to the usual absence of host effects in the tests.

For example, in vitro, antibiotic testing using the broth microdilution method is usually performed with exponentially growing mycobacteria as a suspension under optimal conditions in an aerated nutrient-rich broth, which hardly bears any resemblance with the actual host environment [41]. In addition, there are practical considerations that must be considered. An important fact about NTM is that owing to the intrinsic hydrophobicity of their cell walls; they generally attach to the surface of the individual wells in the 96-well plates rather than staying in the aqueous suspension [136]. Therefore, merely measuring the turbidity of cell suspension can lead to inaccurate results, and results can be different from the ones from cells present in surface-attached biofilms [48]. Although microdilution continues to be the most trusted in vitro method, it does have some drawbacks, which include: a large volume of reagents, long experimental time, the possibility of false positives due to long incubation times, chances of cross-contamination, etc. [137].

Strain selection is a crucial aspect of in vitro testing. Typically, *M. smegmatis* is used for laboratory testing and modeling of NTM disease pathogenesis due to its rapid growth rate and ease of handling [138]. However, most isolates of *M. smegmatis* are derived from the same ATCC 607 strain, a strain that has become “lab adapted” by losing several unique NTM features like slow growth and the cell-wall hydrophobicity. As a result, the susceptibility to specific compounds can be seriously overestimated if only typical lab strains are taken as a reference [48]. This, however, should not completely disregard the use of these strains, as, for example, bedaquiline was discovered by using *M. smegmatis* as a model [139]. Moreover, strains of the same species obtained from different sources can have different growth rates, thus affecting the quality of the model used to study the disease. Similarly, two patients infected with the same strain can have different susceptibility to the same antibiotic because of differences in immunity [140]. It is therefore recommended to validate drug susceptibilities in a panel of NTM isolates to increase the chances of selecting strains with the closest growth profile as the isolate of interest [131,141].

During antibiotic susceptibility testing, it is important to remember that the growth rate of the strain can have a significant impact on its susceptibility towards the drug. For example, *M. avium* bacteria are more susceptible in media that supports faster growth than in nutrient-limited medium [142]. Similarly, it is important to note that the same strain may have different colonial variants, and these can have remarkably different susceptibilities. For instance, in *M. avium*, transparent colonies that are usually obtained from isolates of patients are more antibiotic-resistant than the opaque variant that appear during laboratory cultivation [143]. Therefore, these results must be taken with caution: high susceptibility levels in vitro may not necessarily imply an effective in vivo outcome [144].

Indeed, the discrepancy between in vitro and in vivo results is evidenced by the lack of effect of moxifloxacin in *M. abscessus* in vivo, despite encouraging in vitro results [145]. On the other side, cefoxitin and imipenem showed only moderate in vitro activity against *M. abscessus* but are nonetheless effective in vivo, possibly to the differential testing conditions [94].

### 3.3. In Vivo Models

Following in vitro tests, animal models are used for preclinical in vivo testing. Animal models are used essentially to understand the pathogenesis, host immune responses, and for testing potential antimicrobial compounds and vaccines [146]. Cell-based assays provide restricted information about the absorption, distribution, metabolism, excretion and toxicity of screening compounds, but results obtained from animal models often reveal such insights. Here, a persisting issue is the paucity of suitable animal models available for studying NTM pathology [147,148]. Because of the low virulence of NTM compared with *M. tuberculosis*, it is usually difficult to generate an infection in animals unless they are severely immunocompromised since, for instance, lung pathology can only be observed to some extent [149]. This, however, leads to a complication. Since NTM infection in humans may manifest as localized lung infections (in immunocompetent persons) or disseminated infections (in immunocompromised persons), the animal models should be chosen according to the disease of interest [150]. However, only immunocompromised models can sustain low pathogenic NTM infections and therefore, it is generally very difficult to simulate chronic, localized NTM diseases [146]. The overall consequence of the above problems is inconsistent results that are difficult to reproduce. Ideally, animal models that possess hallmarks of human NTM pathology are required for better in vivo results in the testing of NTM anti-mycobacterials [41].

Apart from the traditional animal models—mice, guinea pigs, and rabbits; the recent development of cellular models as well as using the zebrafish embryos have proven to be useful alternatives [146,147,151]. Depending on various parameters, every animal model has its own advantages and disadvantages and hence, some are more valuable in testing potential antimicrobial compounds than the others [146].

Mouse models are widely used because of the abundance of reagents and their low-cost, and they have been instrumental in understanding the host immune response to tuberculosis [146]. Since there are two major categories of NTM diseases (lung disease and extrapulmonary-disseminated disease), the mouse strain that is chosen depends on the disease of interest, despite the fact that it is difficult to mimic chronic NTM infection, which is exclusively isolated to the lungs [146]. Earlier studies also revealed the most immunocompetent mouse strains, like C57BL/6, serve as excellent models for more virulent *M. avium* complex species, but are cleared when infected with *M. abscessus* [152]. In these studies, C57BL/6 and leptin-deficient (Ob/Ob) mice that were infected with *M. abscessus* (with low-dose aerosol inoculum) did not develop a sustained infection; while on the other hand, when infected with high-dose aerosol inoculum, these mice developed an infection that was subsequently cleared [147,153]. Using severely immunocompromised mice as a model is advantageous due to the presence of foamy cells and necrotizing as well as non-necrotizing granulomas in the lungs, 40 days post-infection, which is observed in histopathologic sections of human NTM lung disease [127]. However, one of the challenges is that most strains are able to clear infections by *M. abscessus* within the first few weeks post-infection, which makes the development and selection of the model extremely challenging [152,153,154].

For studying Buruli ulcers, a chronic NTM infection caused by *M. ulcerans* that infects the skin, soft tissues and bone in humans, mouse models are the most used [155]. Other models, such as guinea pigs, have also been used as models for studying Buruli ulcers and characterizing the pathogenicity of *M. ulcerans* and its mycolactone toxins [156,157,158]. Nevertheless, they are much less commonly used because of their resistance to *M. ulcerans* infections [155].

Zebrafish has successfully been established as an efficient model to study infectious diseases in the last decades [159]. Their embryos offer unique in vivo imaging possibilities due to their transparency, and a high number of existing transgenic reporter lines expressing fluorescent proteins permit tracking various immune cell types while they interact with pathogens [160,161,162,163]. Importantly, zebrafish larvae rely only on innate immune defenses during the first weeks, thus being attractive models to study infections that require the host to be immunosuppressed to a certain degree. Zebrafish models permit high-throughput analysis and therefore are convenient for the initial steps of preclinical evaluation. One of the most studied zebrafish infection models is the zebrafish tuberculosis model. Infections conducted on zebrafish have revised our interpretation of the mechanism of granuloma formation by allowing real-time visualization of the biological events that take place inside the host, in particular the pathogen-macrophage interactions [164]. However, it is important to mention that these models, in most cases, utilize *M. marinum* to simulate *M. tuberculosis* infection, and studies of pathogenesis on this model may be, therefore, applicable to other mycobacteria. Considering the common ancestry of *M. marinum* and M. *ulcerans*, future research could also focus on the difference of specific virulence determinants of these strains, such as the importance of mycolactones that are encoded on plasmids specific for the *M. ulcerans* lineage. In addition to *M. marinum*, pathogenesis models of *M. kansasii* [165] and of *M. abscessus* [166,167] exist in zebrafish, which can be used for high-throughput drug screen processes.

Since most animal models develop disseminated infections instead of localized infections seen in humans, the search for a robust NTM model is not yet complete. Non-human primates have been used as models to study NTM infections because of their closer resemblance to human immunology and physiology. Rhesus macaques have been shown to develop isolated pulmonary infections that tend to persist for long, similar to NTM pathogenesis observed in humans [168]. Similar observations have also been made for marmosets [169]. Nevertheless, in addition to ethical concerns, their availability, purchase and husbandry cost present a practical limitation to their use [170]. Moreover, smaller sample sizes of these animals used in disease studies may lead to statistically insignificant results, and even small genetic changes can cause greater variance [171].

### 3.4. Iterative Approach to Drug Design

**Figure 2:**
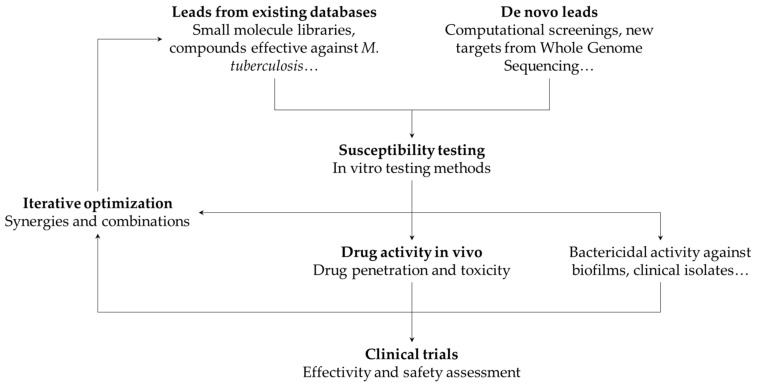
Overview of the development process for new therapies against NTMs.

Despite using different tools and giving different experimental insights, the drug discovery process is usually an iterative optimization of the techniques presented previously (Figure 2). For example, the first step towards the discovery of new antimicrobial therapies is usually the screen of compounds that show potential for inhibiting mycobacterial growth. Since it is not possible to try every compound for activity against NTM, narrowing the search space is important to save time, money, and resources. This can be done in silico, based on drug-target interactions or structure–activity relationship models [172,173,174]. The opposite approach is also viable: libraries of bioactive compounds with unknown effects in NTM infections can be screened in vitro, and the results used to improve computational models and predictions. Similarly, information about in vivo drug pharmacokinetics is an important step during a preclinical study for a new therapy. Understanding system and drug-specific properties and modeling them with systems biology or systems pharmacology models provide important information that potentially can speed up the drug development process [175].

Recently, artificial intelligence methods such as machine learning have been used not only to estimate the bioactivity of drug candidates [176] but also to complement existing tools across different stages in the NTM drug discovery process, for example, in automating the cell count from fluorescence microscopy imaging and identification of mycobacteria species from mass spectroscopy [177,178].

## 4. Considerations for the Design of Therapies against NTMs

### 4.1. Optimization of Known Compounds Relevant for Combatting NTM

When designing new possible therapies against NTMs, it is important to consider the mechanisms that confer drug resistance that we reviewed above. For example, the exceptionally high hydrophobicity of mycobacterial cell walls has an important bearing on drug design: the more lipophilic molecules generally show higher permeability and hence are more active. This means that a possible route to developing anti-NTM antibiotics is to synthesize hydrophobic derivatives of existing antibiotics [179]. For instance, ciprofloxacin, when modified by the addition of hydrophobic alkyl substituents, showed higher activity against *M. avium* [180]. Similarly, for *M. leprae*, the efficacy of fluoroquinolones improved by incorporating the hydrophobic cyclopropyl groups [181].

Compounds that have been identified to be active against other diseases may directly be screened by in vitro bacterial assays, and MIC values may be determined to check the efficiency of the drug [182]. However, for *M. abscessus*, the hit rates among drug libraries that are active against neglected diseases like ascariasis, Buruli ulcer, Chagas disease, and malaria is just 1% [183], highlighting the great difficulty in finding new drugs for NTMs. A way forward could be to screen the compound libraries active against tuberculosis for their effect against NTM because of the structural similarity and homology of their drug targets [184]. In a recently conducted study, 129 compounds known to be active against *M. tuberculosis* were tested against *M. abscessus* and *M. avium,* and their rates were higher than for drugs that are not active against tuberculosis [185]. Rifabutin, an antibiotic used for the treatment of tuberculosis, has recently been found to be active against *M. abscessus* [182]. Notwithstanding these positive outcomes, most existing drugs specific to tuberculosis are usually ineffective against NTM [185,186].

### 4.2. Synergies and Combination Therapies

In the development of a novel treatment, it should be considered that most of the successful anti-NTM drug therapies involve synergistic effects of two drugs: one antibiotic to disrupt the permeability of the outer membrane in order to ensure entry of the drug into the cell, and the another disrupting at least one vital cellular processes (such as DNA, RNA, or protein and outer membrane synthesis) for inhibiting cell growth [48]. For example, the performance of hydrophobic drugs that have intracellular targets can be improved by using them in conjunction with compounds that specifically target cell wall homeostasis. Such synergistic effects have been observed between ethambutol plus rifampicin in *M. avium* [187] or vancomycin plus clarithromycin in *M. abscessus* [188]. Similar effects can be seen using adjuvants that inhibit specific efflux pumps [189,190] or that increase the expression of the enzymes required for the biotransformation of a prodrug, thus boosting antibiotic effectivity [191].

Moreover, the synergistic effects cannot only improve drug efficacy but also reduce the chances that treatments lead to drug resistance [192,193]. For instance, the combination of β-lactam antibiotics and β-lactamase inhibitors has shown to be a promising strategy against *M. avium* infections [89]. Further, synergies can also be achieved by using a combination of two or more drugs that have the same cellular target. This approach has been validated for *M. abscessus* complex, wherein a dual β-lactam drug regimen proved to be much more effective than a single-drug regimen, with or without β-lactamase inhibitors [88,194]. In such regimens, each of the β-lactams preferentially targets a different enzyme that is involved in cell wall synthesis, thereby ensuring that “overlapping” effects are minimized, and combinedly, all biochemical pathways are exhaustively targeted [194]. Typically, mutations that cause the development of resistance mechanisms can have subsequent “spill-over” effects: the same mechanism may confer resistance to the entire class of drugs that target the same biochemical pathway (cross-resistance), or it can lead to increased vulnerability to other drugs that target a different pathway (collateral sensitivity) [195]. Exploiting collateral sensitivity by either combinatorial or cyclical treatment regimens involving multiple drugs can be an effective strategy, as recently demonstrated with drugs directed against *M. marinum* [196].

### 4.3. Host-Directed Therapies

A different approach towards finding more effective treatments against NTM infections is host-directed therapies (HDT), in which specific immune pathways of the host are modulated in such a way that it leads to a better clinical outcome. That is, HDTs aims to empower the host to clear the infection instead of directly targeting bacteria. The ways in which HDTs can help against NTMs are strengthening innate immunity against mycobacterial infections, preventing the growth of the bacilli by inhibiting the essential host-related growth-factors, restoring the immune response suppressed due to the infection, or reducing tissue damage due to hyperinflammation [197,198].

HDTs offer several unique advantages over conventional therapies. First, chances of drug-related resistance are considerably reduced because it is difficult for the bacteria to develop completely new mechanisms of interacting with the host quickly and while being under the same hostile immune selection [199]. They also offer the possibility of making conventional drugs more effective against already resistant strains by neutralizing pathogen defenses [200]. The synergistic role of HDT adjuvants with anti-mycobacterials was demonstrated in a study in which picolinic acid (PA) was shown to potentiate fluoroquinolones against bacteria from the *M. avium* complex [201]. Fluoroquinolones are otherwise only very weakly effective against *M. avium* infections. This was attributed to two factors- upregulation of the immune system by PA and chelation of Fe ions by PA, which deprive the bacteria of the essential ions needed for growth [202]. In a previous study, PA was shown to inhibit *M. avium* growth inside mouse macrophages by inducing apoptosis- causing morphological changes [203].

In addition, some compounds show both host-directed and bacterial-directed actions. Clofazimine, a commonly used drug in *M. leprae* infections, is a good example of a drug that simultaneously affects the host as well as the bacteria. Upon infecting the body, *M. leprae* creates a safe microenvironment for itself inside the macrophages of the host by increasing the accumulation and retarding the breakdown of macrophage lipids [204]. It was shown that clofazimine not only helps reverse these two processes but also activates immune reactions in *M. leprae* infected host cells. Hence, effectively, it not just prevents the growth of the bacteria but also actively helps to eliminate it [205]. Another example of an antibiotic with a strong effect on the host inflammatory system is minocycline that modulates the endocannabinoid signaling pathway and, in this way, might have HDT potential [206].

Finally, considering the mechanisms of defense of the host system can lead to more effective ways of delivering drugs to the target, adding value to existing treatments. An example would be precision-targeting the drug by loading them into the host cells that act as carriers. This approach was demonstrated by loading dendritic cells to deliver amikacin inside alveolar granulomas and thus enhancing the killing of residing mycobacteria [207].

However, a major challenge in the development of effective HDTs is that different patients may not have the same immune status, which may depend on factors like the stage of the disease, health of the individual, pre-existing conditions, and genetic makeup [200]. This can be a hindrance towards a universal HDT and may require an approach for personalized medicine, much like cancer immunotherapy [208].

## 5. Summary and Future Perspectives

The discovery and validation for new therapies against NTMs is an urgent necessity as increasing cases of these infections are being reported worldwide, and existing therapies prove to be ineffective. We discussed the reasons why the development of effective antimycobacterial drugs remains elusive: their intrinsic resistance against antimicrobial compounds. Several molecular mechanisms are used by mycobacteria to survive current antibiotic therapies, including a thick impermeable hydrophobic cell wall that acts as the first line of defense; intracellular enzymes that reduce the antimicrobial effect of the drug; efflux pumps that expel molecules from the cytoplasm; and adaptive mechanisms that prevent drugs from sequestering its target. In addition, even if the drug is effective in killing mycobacteria or inhibiting their growth, mycobacterial colonies can persist on surfaces by forming inert biofilms or enter latent states within the granulomas inside the host.

We delineated the main techniques and models that can be used for the development of new effective therapies against NTM and how they can complement each other in different stages of the drug development pipeline, thus accelerating drug discovery. For example, whole-genome sequencing can provide crucial leads in target identification based on the genetic makeup of the strains, which can be followed by in silico drug-target interaction studies to identify the potential drug molecules that can effectively dock on the target and initiate action. Validation of these drugs and the determination of their efficacy would require testing on clinical isolates, taking into account variations arising due to different colonial morphologies, and media-dependent growth rates, among others. To fully understand the mechanism of drug action, suitable animal models are very important. Moreover, insights on in vivo infection growth can help to select the relevant drug regimes that focus on the specific touchpoints of the mechanism, rather than a general broad-spectrum therapy. Finally, it is important to mention that when dealing with mycobacteria, an effective drug regime would include not only one drug but must work in combination with other antimicrobials or host-directed therapies, thus improving drug activity while preventing the buildup of resistance against any single drug.

## Figures and Tables

**Table 1:** Summary of the nontuberculous mycobacteria (NTMs) mentioned in this review, their classification according to Runyon, and their reported pathogenesis in humans.

Runyon Classification	NTM Species	Pathogenesis in Humans
Photochromogens Runyon type I	*M. kansasii* [16], *M. simiae* [17]	Pulmonary infections Skin infections Disseminated infections
	*M. marinum* [18]	Skin and soft tissue infections Disseminated infections
Scotochromogens Runyon type II	*M. gordonae* [19]	Pulmonary infections Skin infections Disseminated infections
	*M. scrofulaceum* [20]	Cervical lymphadenitis among children Pulmonary infections Disseminated infections
Non-photochromogens Runyon type III	*M. avium* complex (*M. avium* and *M. intracellulare*) [21]	Pulmonary MAC infections Disseminated infections (mostly in AIDS patients) MAC associated lymphadenitis (in young kids and people with normal immune systems)
	*M. malmoense* [22]	Pulmonary infections Disseminated infections
	*M. ulcerans* [18]	Skin diseases (Buruli ulcers)
Rapid growing Runyon type IV	*M. abscessus* [23]	Pulmonary infections Skin and Soft tissue disease Central nervous system infections Disseminated infections
	*M. chelonae* [24]	Skin and soft tissue infections Pulmonary infections Disseminated infections
	*M. smegmatis*	Widely regarded as nonpathogenic

**Table 2:** Summary of the current methods available for the discovery of new antimicrobial therapies in NTMs.

	In Silico	In Vitro	In Vivo
**Methods employed**	Structure–activity relations, Molecular simulations, Comparative genomics	Antimicrobial effect tests on cultured cells	Tests on live infected animals
**Main insights**	Molecular basis for drug action	Molecular and cellular effect of drug action	Whole-organism level of drug action
**Advantages**	High throughput, low-cost, no need for actual chemical synthesis of compounds or bacterial growth	Relatively simple systems and lower cost and time involvement, easy to handle, scalable	Closer to the actual physiological environment
**Limitations**	Requires prior information and complicated models to simulate molecular events such as docking and drug-target interactions	Needs a high level of standardization and careful experimentation for reproducibility, may not reproduce clinical situations	Requires careful model selection, large organism response is less predictable, ethical considerations, high economical costs
**Best-fit stage in drug discovery**	Primary (for narrowing the search of potential candidates) or secondary (for optimizing compounds to species-specific targets)	Secondary (for screening initial targets and efficacy determination)	Tertiary (for preclinical evaluation)

## Data Availability

Not applicable.

## References

[1] Global Tuberculosis Report 2020. https://www.who.int/teams/global-tuberculosis-programme/tb-reports.

[2] Leprosy. https://www.who.int/news-room/fact-sheets/detail/leprosy.

[3] Yates V.M. (2010). Mycobacterial Infections. Rook’s Textbook of Dermatology.

[4] Griffith D.E., Aksamit T., Brown-Elliott B.A., Catanzaro A., Daley C., Gordin F., Holland S.M., Horsburgh R., Huitt G., Iademarco M.F. (2007). An official ATS/IDSA Statement: Diagnosis, treatment, and prevention of nontuberculous mycobacterial diseases. Am. J. Respir. Crit. Care Med..

[5] Kendall B., Winthrop K. (2013). Update on the epidemiology of pulmonary nontuberculous mycobacterial infections. Semin. Respir. Crit. Care Med..

[6] Ratnatunga C.N., Lutzky V.P., Kupz A., Doolan D.L., Reid D.W., Field M., Bell S.C., Thomson R.M., Miles J.J. (2020). The rise of non-tuberculosis mycobacterial lung disease. Front. Immunol..

[7] Gopalaswamy R., Shanmugam S., Mondal R., Subbian S. (2020). Of tuberculosis and non-tuberculous mycobacterial infections–a comparative analysis of epidemiology, diagnosis and treatment. J. Biomed. Sci..

[8] Runyon E.H. (1959). Anonymous mycobacteria in pulmonary disease. Med. Clin. N. Am..

[9] Kim C.-J., Kim N.-H., Song K.-H., Choe P.G., Kim E.S., Park S.W., Kim H.-B., Kim N.-J., Kim E.-C., Park W.B. (2013). Differentiating rapid- and slow-growing mycobacteria by difference in time to growth detection in liquid media. Diagn. Microbiol. Infect. Dis..

[10] Porvaznik I., Solovič I., Mokrý J. (2017). Non-tuberculous mycobacteria: Classification, diagnostics, and therapy. Adv. Exp. Med. Biol..

[11] Johnson M.M., Odell J.A. (2014). Nontuberculous mycobacterial pulmonary infections. J. Thorac. Dis..

[12] Nessar R., Cambau E., Reyrat J.M., Murray A., Gicquel B. (2012). Mycobacterium abscessus: A new antibiotic nightmare. J. Antimicrob. Chemother..

[13] Devulder G., de Montclos M.P., Flandrois J.P. (2005). A multigene approach to phylogenetic analysis using the genus Mycobacterium as a model. Int. J. Syst. Evol. Microbiol..

[14] Gupta R.S., Lo B., Son J. (2018). Phylogenomics and comparative genomic studies robustly support division of the genus mycobacterium into an emended genus mycobacterium and four novel genera. Front. Microbiol..

[15] Wee W.Y., Dutta A., Choo S.W. (2017). Comparative genome analyses of mycobacteria give better insights into their evolution. PLoS ONE.

[16] Jagielski T., Borówka P., Bakuła Z., Lach J., Marciniak B., Brzostek A., Dziadek J., Dziurzyński M., Pennings L., van Ingen J. (2020). Genomic insights into the mycobacterium kansasii complex: An update. Front. Microbiol..

[17] Hamieh A., Tayyar R., Tabaja H., Zein S.E.L., Bou Khalil P., Kara N., Kanafani Z.A., Kanj N., Bou Akl I., Araj G. (2018). Emergence of *Mycobacterium simiae*: A retrospective study from a tertiary care center in Lebanon. PLoS ONE.

[18] Franco-Paredes C., Marcos L.A., Henao-Martínez A.F., Rodríguez-Morales A.J., Villamil-Gómez W.E., Gotuzzo E., Bonifaz A. (2018). *Cutaneous Mycobacterial* infections. Clin. Microbiol. Rev..

[19] Douglas J.G., Calder M.A., Choo-Kang Y.F.J., Leitch A.G. (1986). *Mycobacterium gordonae*: A new pathogen?. Thorax.

[20] Suzuki S., Morino E., Ishii M., Namkoong H., Yagi K., Asakura T., Asami T., Fujiwara H., Uwamino Y., Nishimura T. (2016). Clinical characteristics of pulmonary *Mycobacterium scrofulaceum* disease in 2001-2011: A case series and literature review. J. Infect. Chemother..

[21] Han X.Y., Tarrand J.J., Infante R., Jacobson K.L., Truong M. (2005). Clinical significance and epidemiologic analyses of *Mycobacterium avium* and *Mycobacterium intracellulare* among patients without AIDS. J. Clin. Microbiol..

[22] Doig C., Muckersie L., Watt B., Forbes K.J. (2002). Molecular epidemiology of *Mycobacterium malmoense* infections in Scotland. J. Clin. Microbiol..

[23] To K., Cao R., Yegiazaryan A., Owens J., Venketaraman V. (2020). General Overview of nontuberculous mycobacteria opportunistic pathogens: *Mycobacterium avium* and *Mycobacterium abscessus*. J. Clin. Med..

[24] Jones R.S., Shier K.L., Master R.N., Bao J.R., Clark R.B. (2019). Current significance of the *Mycobacterium chelonae*-abscessus group. Diagn. Microbiol. Infect. Dis..

[25] Máiz Carro L., Barbero Herranz E., Nieto Royo R. (2018). Respiratory infections due to nontuberculous mycobacterias. Med. Clin..

[26] Nishiuchi Y., Iwamoto T., Maruyama F. (2017). Infection sources of a common non-tuberculous mycobacterial pathogen, *Mycobacterium avium* complex. Front. Med..

[27] Shah N.M., Davidson J.A., Anderson L.F., Lalor M.K., Kim J., Thomas H.L., Lipman M., Abubakar I. (2016). Pulmonary *Mycobacterium avium*-intracellulare is the main driver of the rise in non-tuberculous mycobacteria incidence in England, Wales and Northern Ireland, 2007–2012. BMC Infect. Dis..

[28] Johansen M.D., Herrmann J.L., Kremer L. (2020). Non-tuberculous mycobacteria and the rise of *Mycobacterium abscessus*. Nat. Rev. Microbiol..

[29] Fleshner M., Olivier K.N., Shaw P.A., Adjemian J., Strollo S., Claypool R.J., Folio L., Zelazny A., Holland S.M., Prevots D.R. (2016). Mortality among patients with pulmonary non-tuberculous mycobacteria disease. Int. J. Tuberc. Lung Dis..

[30] Jarand J., Levin A., Zhang L., Huitt G., Mitchell J.D., Daley C.L. (2011). Clinical and microbiologic outcomes in patients receiving treatment for *Mycobacterium abscessus* pulmonary disease. Clin. Infect. Dis..

[31] Taylor J.L., Palmer S.M. (2006). *Mycobacterium abscessus* chest wall and pulmonary infection in a cystic fibrosis lung transplant recipient. J. Hear. Lung Transplant..

[32] Bryant J.M., Grogono D.M., Rodriguez-Rincon D., Everall I., Brown K.P., Moreno P., Verma D., Hill E., Drijkoningen J., Gilligan P. (2016). Emergence and spread of a humantransmissible multidrug-resistant nontuberculous mycobacterium. Science.

[33] Hermansen T.S., Ravn P., Svensson E., Lillebaek T. (2017). Nontuberculous mycobacteria in Denmark, incidence and clinical importance during the last quarter-century. Sci. Rep..

[34] Baldwin S.L., Larsenid S.E., Ordway D., Cassell G., Coler R.N. (2019). The complexities and challenges of preventing and treating nontuberculous mycobacterial diseases. PLoS Negl. Trop. Dis..

[35] Faverio P., Stainer A., Bonaiti G., Zucchetti S.C., Simonetta E., Lapadula G., Marruchella A., Gori A., Blasi F., Codecasa L. (2016). Characterizing non-tuberculous mycobacteria infection in bronchiectasis. Int. J. Mol. Sci..

[36] Santin M., Dorca J., Alcaide F., Gonzalez L., Casas S., Lopez M., Guerra M.R. (2009). Long-term relapses after 12-month treatment for *Mycobacterium kansasii* lung disease. Eur. Respir. J..

[37] Sim Y.S., Park H.Y., Jeon K., Suh G.Y., Kwon O.J., Koh W.-J. (2010). Standardized combination antibiotic treatment of *Mycobacterium avium* complex lung disease. Yonsei Med. J..

[38] Wallace R.J., Dukart G., Brown-Elliott B.A., Griffith D.E., Scerpella E.G., Marshall B. (2014). Clinical experience in 52 patients with tigecycline-containing regimens for salvage treatment of *Mycobacterium abscessus* and *Mycobacterium chelonae* infections. J. Antimicrob. Chemother..

[39] (1997). Diagnosis and treatment of disease caused by nontuberculous mycobacteria. This official statement of the American Thoracic Society was approved by the Board of Directors, March 1997. Medical Section of the American Lung Association. Am. J. Respir. Crit. Care Med..

[40] Mirsaeidi M., Farshidpour M., Allen M.B., Ebrahimi G., Falkinham J.O. (2014). Highlight on advances in nontuberculous mycobacterial disease in North America. Biomed Res. Int..

[41] Wu M.-L., Aziz D.B., Dartois V., Dick T. (2018). NTM drug discovery: Status, gaps and the way forward. Drug Discov. Today.

[42] Munita J.M., Arias C.A., Unit A.R., Santiago A. (2016). De HHS Public access mechanisms of antibiotic resistance. HHS Public Access.

[43] Moon S.M., Park H.Y., Kim S.-Y., Jhun B.W., Lee H., Jeon K., Kim D.H., Huh H.J., Ki C.-S., Lee N.Y. (2016). Clinical characteristics, treatment outcomes, and resistance mutations associated with macrolide-resistant *Mycobacterium avium* complex lung disease. Antimicrob. Agents Chemother..

[44] Nasiri M.J., Haeili M., Ghazi M., Goudarzi H., Pormohammad A., Imani Fooladi A.A., Feizabadi M.M. (2017). New insights in to the intrinsic and acquired drug resistance mechanisms in mycobacteria. Front. Microbiol..

[45] Hayes J.D., Wolf C.R. (1990). Molecular mechanisms of drug resistance. Biochem. J..

[46] Jarlier V., Nikaido H. (1994). Mycobacterial cell wall: Structure and role in natural resistance to antibiotics. FEMS Microbiol. Lett..

[47] Falkinham J.O. (2007). Growth in catheter biofilms and antibiotic resistance of *Mycobacterium avium*. J. Med. Microbiol..

[48] Falkinham J.O. (2018). Challenges of NTM drug development. Front. Microbiol..

[49] Helguera-Repetto A.C., Chacon-Salinas R., Cerna-Cortes J.F., Rivera-Gutierrez S., Ortiz-Navarrete V., Estrada-Garcia I., Gonzalez-y-Merchand J.A. (2014). Differential macrophage response to slow- and fast-growing pathogenic mycobacteria. Biomed. Res. Int..

[50] Ren H., Liu J. (2006). AsnB is involved in natural resistance of *Mycobacterium smegmatis* to multiple drugs. Antimicrob. Agents Chemother..

[51] Wolff K.A., Nguyen H.T., Cartabuke R.H., Singh A., Ogwang S., Nguyen L. (2009). Protein Kinase G Is required for intrinsic antibiotic resistance in mycobacteria. Antimicrob. Agents Chemother..

[52] Cangelosi G.A., Palermo C.O., Laurent J.-P., Hamlin A.M., Brabant W.H. (1999). Colony morphotypes on Congo red agar segregate along species and drug susceptibility lines in the *Mycobacterium avium-intracellulare* complex. Microbiology.

[53] Cangelosi G.A., Do J.S., Freeman R., Bennett J.G., Semret M., Behr M.A. (2006). The two-component regulatory system mtrAB is required for morphotypic multidrug resistance in *Mycobacterium avium*. Antimicrob. Agents Chemother..

[54] Philalay J.S., Palermo C.O., Hauge K.A., Rustad T.R., Cangelosi G.A. (2004). Genes required for intrinsic multidrug resistance in *Mycobacterium avium*. Antimicrob. Agents Chemother..

[55] Smith T., Wolff K.A., Nguyen L. (2013). Molecular biology of drug resistance in *Mycobacterium tuberculosis*. Curr. Top. Microbiol. Immunol..

[56] Nguyen L., Chinnapapagari S., Thompson C.J. (2005). FbpA-dependent biosynthesis of trehalose dimycolate is required for the intrinsic multidrug resistance, cell wall structure, and colonial morphology of *Mycobacterium smegmatis*. J. Bacteriol..

[57] Niederweis M. (2008). Nutrient acquisition by mycobacteria. Microbiology.

[58] Sharbati S., Schramm K., Rempel S., Wang H., Andrich R., Tykiel V., Kunisch R., Lewin A. (2009). Characterisation of porin genes from *Mycobacterium fortuitum* and their impact on growth. BMC Microbiol..

[59] Lambert P.A. (2002). Cellular impermeability and uptake of biocides and antibiotics in Gram-positive bacteria and mycobacteria. J. Appl. Microbiol..

[60] Sharbati-Tehrani S., Stephan J., Holland G., Appel B., Niederweis M., Lewin A. (2005). Porins limit the intracellular persistence of *Mycobacterium smegmatis*. Microbiology.

[61] Stephan J., Mailaender C., Etienne G., Daffeé M., Niederweis M. (2004). Multidrug resistance of a porin deletion mutant of *Mycobacterium smegmatis*. Antimicrob. Agents Chemother..

[62] Danilchanka O., Pavlenok M., Niederweis M. (2008). Role of porins for uptake of antibiotics by *Mycobacterium smegmatis*. Antimicrob. Agents Chemother..

[63] Falkinham J.O. (2011). Nontuberculous mycobacteria from household plumbing of patients with nontuberculous mycobacteria disease. Emerg. Infect. Dis..

[64] Van der Wielen P.W.J.J., van der Kooij D. (2013). Nontuberculous mycobacteria, fungi, and opportunistic pathogens in unchlorinated drinking water in The Netherlands. Appl. Environ. Microbiol..

[65] Kostakioti M., Hadjifrangiskou M., Hultgren S.J. (2013). Bacterial biofilms: Development, dispersal, and therapeutic strategies in the dawn of the postantibiotic era. Cold Spring Harb. Perspect. Med..

[66] Yamazaki Y., Danelishvili L., Wu M., Hidaka E., Katsuyama T., Stang B., Petrofsky M., Bildfell R., Bermudez L.E. (2006). The ability to form biofilm influences *Mycobacterium avium* invasion and translocation of bronchial epithelial cells. Cell. Microbiol..

[67] Carter G., Wu M., Drummond D.C., Bermudez L.E. (2003). Characterization of biofilm formation by clinical isolates of *Mycobacterium avium*. J. Med. Microbiol..

[68] Brodlie M., Aseeri A., Lordan J.L., Robertson A.G.N., McKean M.C., Corris P.A., Griffin S.M., Manning N.J., Pearson J.P., Ward C. (2015). Bile acid aspiration in people with cystic fibrosis before and after lung transplantation. Eur. Respir. J..

[69] Simoes M. (2011). Antimicrobial strategies effective against infectious bacterial biofilms. Curr. Med. Chem..

[70] Faria S., Joao I., Jordao L. (2015). General overview on nontuberculous mycobacteria, biofilms, and human Infection. J. Pathog..

[71] Mah T.-F. (2012). Biofilm-specific antibiotic resistance. Future Microbiol..

[72] Casadevall A., Pirofski L. (2009). Virulence factors and their mechanisms of action: The view from a damage–response framework. J. Water Health.

[73] Steed K.A., Falkinham J.O. (2006). Effect of growth in biofilms on chlorine susceptibility of *Mycobacterium avium* and *Mycobacterium intracellulare*. Appl. Environ. Microbiol..

[74] Ehlers S., Schaible U.E. (2013). The granuloma in tuberculosis: Dynamics of a host–pathogen collusion. Front. Immunol..

[75] Gonzalez-Juarrero M., Turner O.C., Turner J., Marietta P., Brooks J.V., Orme I.M. (2001). Temporal and spatial arrangement of lymphocytes within lung granulomas induced by aerosol infection with *Mycobacterium tuberculosis*. Infect. Immun..

[76] Puissegur M.-P., Botanch C., Duteyrat J.-L., Delsol G., Caratero C., Altare F. (2004). An in vitro dual model of mycobacterial granulomas to investigate the molecular interactions between mycobacteria and human host cells. Cell. Microbiol..

[77] Zhang L., Lin W.M., Li H., Dai X.D., Ma S.P., Ren W.H., Jeon S.K., Lee J.M. (2019). Hepatic nontuberculous mycobacterial granulomas in patients with cancer mimicking metastases: An analysis of three cases. Quant. Imaging Med. Surg..

[78] Ufimtseva E. (2015). Mycobacterium -host cell relationships in granulomatous lesions in a mouse model of latent tuberculous infection. Biomed Res. Int..

[79] Dutta N.K., Karakousis P.C. (2014). Latent tuberculosis infection: Myths, models, and molecular mechanisms. Microbiol. Mol. Biol. Rev..

[80] Machado D., Lecorche E., Mougari F., Cambau E., Viveiros M. (2018). Insights on *Mycobacterium leprae* Efflux Pumps and their implications in drug resistance and virulence. Front. Microbiol..

[81] Li X.-Z., Zhang L., Nikaido H. (2004). Efflux pump-mediated intrinsic drug resistance in *Mycobacterium smegmatis*. Antimicrob. Agents Chemother..

[82] Liu J., Takiff H.E., Nikaido H. (1996). Active efflux of fluoroquinolones in *Mycobacterium smegmatis* mediated by LfrA, a multidrug efflux pump. J. Bacteriol..

[83] De Rossi E., Blokpoel M.C.J., Cantoni R., Branzoni M., Riccardi G., Young D.B., De Smet K.A.L., Ciferri O. (1998). Molecular cloning and functional analysis of a novel tetracycline resistance determinant, tet(V), from *Mycobacterium smegmatis*. Antimicrob. Agents Chemother..

[84] Silva P.E.A., Bigi F., de la Paz Santangelo M., Romano M.I., Martín C., Cataldi A., Aínsa J.A. (2001). Characterization of P55, a multidrug efflux pump in *Mycobacterium bovis* and *Mycobacterium tuberculosis*. Antimicrob. Agents Chemother..

[85] Kwon H.H., Tomioka H., Saito H. (1995). Distribution and characterization of β-lactamases of mycobacteria and related organisms. Tuber. Lung Dis..

[86] Rominski A., Schulthess B., Müller D.M., Keller P.M., Sander P. (2017). Effect of β-lactamase production and β-lactam instability on MIC testing results for *Mycobacterium abscessus*. J. Antimicrob. Chemother..

[87] Lavollay M., Dubée V., Heym B., Herrmann J.-L., Gaillard J.-L., Gutmann L., Arthur M., Mainardi J.-L. (2014). In vitro activity of cefoxitin and imipenem against *Mycobacterium abscessus* complex. Clin. Microbiol. Infect..

[88] Pandey R., Chen L., Manca C., Jenkins S., Glaser L., Vinnard C., Stone G., Lee J., Mathema B., Nuermberger E.L. (2019). Dual β-lactam combinations highly active against *Mycobacterium abscessus* complex in vitro. MBio.

[89] Lefebvre A.-L., Dubée V., Cortes M., Dorchêne D., Arthur M., Mainardi J.-L. (2016). Bactericidal and intracellular activity of β-lactams against *Mycobacterium abscessus*. J. Antimicrob. Chemother..

[90] Adjei M.D., Heinze T.M., Deck J., Freeman J.P., Williams A.J., Sutherland J.B. (2007). Acetylation and nitrosation of ciprofloxacin by environmental strains of mycobacteria. Can. J. Microbiol..

[91] Payton M., Auty R., Delgoda R., Everett M., Sim E. (1999). Cloning and characterization of arylamine N -acetyltransferase genes from *Mycobacterium smegmatis* and *Mycobacterium tuberculosis*: Increased expression results in isoniazid resistance. J. Bacteriol..

[92] Aínsa J.A., Pérez E., Pelicic V., Berthet F., Gicquel B., Martín C. (1997). Aminoglycoside 2′- N -acetyltransferase genes are universally present in mycobacteria: Characterization of the aac(2′)-Ic gene from *Mycobacterium tuberculosis* and the aac(2 ′ )-Id gene from *Mycobacterium smegmatis*. Mol. Microbiol..

[93] Ripoll F., Pasek S., Schenowitz C., Dossat C., Barbe V., Rottman M., Macheras E., Heym B., Herrmann J.-L., Daffé M. (2009). Non mycobacterial virulence genes in the genome of the emerging pathogen *Mycobacterium abscessus*. PLoS ONE.

[94] Rominski A., Roditscheff A., Selchow P., Böttger E.C., Sander P. (2017). Intrinsic rifamycin resistance of Mycobacterium abscessus is mediated by ADP-ribosyltransferase MAB_0591. J. Antimicrob. Chemother..

[95] Baysarowich J., Koteva K., Hughes D.W., Ejim L., Griffiths E., Zhang K., Junop M., Wright G.D. (2008). Rifamycin antibiotic resistance by ADP-ribosylation: Structure and diversity of Arr. Proc. Natl. Acad. Sci. USA.

[96] Blair J.M.A., Webber M.A., Baylay A.J., Ogbolu D.O., Piddock L.J.V. (2015). Molecular mechanisms of antibiotic resistance. Nat. Rev. Microbiol..

[97] Adizie J., Qasim M., Pagaria M. (2016). S39 Risk of NTM (non tuberculous mycobacterium) infection in patients on long term prophylactic macrolide antibiotics. Thorax.

[98] Meier A., Kirschner P., Springer B., Steingrube V.A., Brown B.A., Wallace R.J., Böttger E.C. (1994). Identification of mutations in 23S rRNA gene of clarithromycin-resistant *Mycobacterium intracellulare*. Antimicrob. Agents Chemother..

[99] Bastian S., Veziris N., Roux A.L., Brossier F., Gaillard J.L., Jarlier V., Cambau E. (2011). Assessment of clarithromycin susceptibility in strains belonging to the *Mycobacterium abscessus* group by erm(41) and rrl sequencing. Antimicrob. Agents Chemother..

[100] Sander P., Prammananan T., Meier A., Frischkorn K., Böttger E.C. (1997). The role of ribosomal RNAs in macrolide resistance. Mol. Microbiol..

[101] Brown-Elliott B.A., Nash K.A., Wallace R.J. (2012). Antimicrobial susceptibility testing, drug resistance mechanisms, and therapy of infections with nontuberculous mycobacteria. Clin. Microbiol. Rev..

[102] Obata S., Zwolska Z., Toyota E., Kudo K., Nakamura A., Sawai T., Kuratsuji T., Kirikae T. (2006). Association of rpoB mutations with rifampicin resistance in *Mycobacterium avium*. Int. J. Antimicrob. Agents.

[103] Klein J.L., Brown T.J., French G.L. (2001). Rifampin resistance in *Mycobacterium kansasii* is associated with rpoB mutations. Antimicrob. Agents Chemother..

[104] Zhu J.-H., Wang B.-W., Pan M., Zeng Y.-N., Rego H., Javid B. (2018). Rifampicin can induce antibiotic tolerance in mycobacteria via paradoxical changes in rpoB transcription. Nat. Commun..

[105] Dey A., Verma A.K., Chatterji D. (2010). Role of an RNA polymerase interacting protein, MsRbpA, from *Mycobacterium smegmatis* in phenotypic tolerance to rifampicin. Microbiology.

[106] Rampacci E., Stefanetti V., Passamonti F., Henao-Tamayo M. (2020). Preclinical models of nontuberculous mycobacteria infection for early drug discovery and vaccine research. Pathogens.

[107] Iskar M., Zeller G., Zhao X.-M., van Noort V., Bork P. (2012). Drug discovery in the age of systems biology: The rise of computational approaches for data integration. Curr. Opin. Biotechnol..

[108] Khurshid Ahmad M.H. (2014). Drug discovery and in silico techniques: A mini-review. Enzym. Eng..

[109] Amberg A. (2013). In Silico Methods. Drug Discovery and Evaluation: Safety and Pharmacokinetic Assays.

[110] Zloh M., Kirton S.B. (2018). The benefits of in silico modeling to identify possible small-molecule drugs and their off-target interactions. Future Med. Chem..

[111] Truman R.W., Ebenezer G.J., Pena M.T., Sharma R., Balamayooran G., Gillingwater T.H., Scollard D.M., McArthur J.C., Rambukkana A. (2014). The armadillo as a model for peripheral neuropathy in leprosy. ILAR J..

[112] Nisha J., Shanthi V. (2015). Computational simulation techniques to understand rifampicin resistance mutation (S425L) of rpoB in *M. leprae*. J. Cell. Biochem..

[113] Vedithi S.C., Lavania M., Kumar M., Kaur P., Turankar R.P., Singh I., Nigam A., Sengupta U. (2015). A report of rifampin-resistant leprosy from northern and eastern India: Identification and in silico analysis of molecular interactions. Med. Microbiol. Immunol..

[114] Nisha J., Shanthi V. (2018). Characterization of ofloxacin interaction with mutated (A91V) Quinolone resistance determining region of DNA gyrase in *Mycobacterium Leprae* through computational simulation. Cell Biochem. Biophys..

[115] Macalino S.J.Y., Billones J.B., Organo V.G., Carrillo M.C.O. (2020). In Silico strategies in tuberculosis drug discovery. Molecules.

[116] Sugiki T., Furuita K., Fujiwara T., Kojima C. (2018). Current NMR techniques for structure-based drug discovery. Molecules.

[117] Batool M., Ahmad B., Choi S. (2019). A structure-based drug discovery paradigm. Int. J. Mol. Sci..

[118] Rehna E.A.A., Singh S.K., Dharmalingam K. (2008). Functional insights by comparison of modeled structures of 18kDa small heat shock protein and its mutant in *Mycobacterium leprae*. Bioinformation.

[119] Ramis I.B., Vianna J.S., Silva Junior L., von Groll A., Ramos D.F., Lobo M.M., Zanatta N., Viveiros M., da Silva P.E.A. (2019). In silico and in vitro evaluation of tetrahydropyridine compounds as efflux inhibitors in *Mycobacterium abscessus*. Tuberculosis.

[120] Sotriffer C.A. (2006). Molecular dynamics simulations in drug design. Encyclopedic Reference of Genomics and Proteomics in Molecular Medicine.

[121] Kumar A., Sechi L.A., Caboni P., Marrosu M.G., Atzori L., Pieroni E. (2015). Dynamical insights into the differential characteristics of *Mycobacterium avium* subsp. *paratuberculosis* peptide binding to HLA-DRB1 proteins associated with multiple sclerosis. New J. Chem..

[122] Ferreira L.L.G., Andricopulo A.D. (2018). Editorial: Chemoinformatics approaches to structure- and ligand-based drug design. Front. Pharmacol..

[123] Ruyck J., Dupont C., Lamy E., Le Moigne V., Biot C., Guérardel Y., Herrmann J., Blaise M., Grassin-Delyle S., Kremer L. (2020). Structure-based design and synthesis of piperidinol-containing molecules as new *Mycobacterium abscessus* inhibitors. ChemistryOpen.

[124] Dupont C., Viljoen A., Dubar F., Blaise M., Bernut A., Pawlik A., Bouchier C., Brosch R., Guérardel Y., Lelièvre J. (2016). A new piperidinol derivative targeting mycolic acid transport in *Mycobacterium abscessus*. Mol. Microbiol..

[125] Bakour S., Sankar S.A., Rathored J., Biagini P., Raoult D., Fournier P.-E. (2016). Identification of virulence factors and antibiotic resistance markers using bacterial genomics. Future Microbiol..

[126] Le Moigne V., Belon C., Goulard C., Accard G., Bernut A., Pitard B., Gaillard J.-L., Kremer L., Herrmann J.-L., Blanc-Potard A.-B. (2016). MgtC as a Host-induced factor and vaccine candidate against *Mycobacterium abscessus* infection. Infect. Immun..

[127] Soni I., De Groote M.A., Dasgupta A., Chopra S. (2016). Challenges facing the drug discovery pipeline for non-tuberculous mycobacteria. J. Med. Microbiol..

[128] Quan T.P., Bawa Z., Foster D., Walker T., Del Ojo Elias C., Rathod P., Iqbal Z., Bradley P., Mowbray J., MMM Informatics Group (2018). Evaluation of whole-genome sequencing for mycobacterial species identification and drug susceptibility testing in a clinical setting: A large-scale prospective assessment of performance against line probe assays and phenotyping. J. Clin. Microbiol..

[129] Matsumoto Y., Kinjo T., Motooka D., Nabeya D., Jung N., Uechi K., Horii T., Iida T., Fujita J., Nakamura S. (2019). Comprehensive subspecies identification of 175 nontuberculous mycobacteria species based on 7547 genomic profiles. Emerg. Microbes Infect..

[130] National Research Council (2000). Summary of Advantages and Disadvantages of in vitro and in vivo methods. Monoclonal Antibody Production.

[131] Jonkman J.H., van Bork L.E., Wijsbeek J., de Zeeuw R.A., Orie N.G., Cox H.L. (1976). “First pass effect” after rectal administration of thiazinamium methylsulphate [proceedings]. J. Pharm. Pharmacol..

[132] Bose M., Venugopal D., Kumar S., Isa M. (2007). Drug resistance profile of human *Mycobacterium avium* complex strains from India. Indian J. Med. Microbiol..

[133] Wallace R.J., Dalovisio J.R., Pankey G.A. (1979). Disk diffusion testing of susceptibility of *Mycobacterium fortuitum* and *Mycobacterium chelonei* to antibacterial agents. Antimicrob. Agents Chemother..

[134] Nair D., Verma J., Rawat D., Hasan A., Capoor M., Gupta K., Deb M., Aggarwal P. (2010). The use of E-test for the drug susceptibility testing of *Mycobacterium tuberculosis*-A solution or an illusion?. Indian J. Med. Microbiol..

[135] Freixo I.M., Caldas P.C.S., Martins F., Brito R.C., Ferreira R.M.C., Fonseca L.S., Saad M.H.F. (2002). Evaluation of etest strips for rapid susceptibility testing of *Mycobacterium tuberculosis*. J. Clin. Microbiol..

[136] Falkinham J.O., Macri R.V., Maisuria B.B., Actis M.L., Sugandhi E.W., Williams A.A., Snyder A.V., Jackson F.R., Poppe M.A., Chen L. (2012). Antibacterial activities of dendritic amphiphiles against nontuberculous mycobacteria. Tuberculosis.

[137] Khan Z.A., Siddiqui M.F., Park S. (2019). Current and emerging methods of antibiotic susceptibility testing. Diagnostics.

[138] Malhotra S., Vedithi S.C., Blundell T.L. (2017). Decoding the similarities and differences among mycobacterial species. PLoS Negl. Trop. Dis..

[139] Andries K., Verhasselt P., Guillemont J., Göhlmann H.W.H., Neefs J.-M., Winkler H., Van Gestel J., Timmerman P., Zhu M., Lee E. (2005). A diarylquinoline drug active on the ATP synthase of *Mycobacterium tuberculosis*. Science.

[140] Von Reyn C.F., Jacobs N.J., Arbeit R.D., Maslow J.N., Niemczyk S. (1995). Polyclonal Mycobacterium avium infections in patients with AIDS: Variations in antimicrobial susceptibilities of different strains of *M. avium* isolated from the same patient. J. Clin. Microbiol..

[141] Van Wijk R.C., Sar A.M., Krekels E.H.J., Verboom T., Spaink H.P., Simonsson U.S.H., Graaf P.H. (2020). Quantification of natural growth of two strains of *Mycobacterium Marinum* for translational antituberculosis drug development. Clin. Transl. Sci..

[142] Falkinham J.O. (2003). Factors influencing the chlorine susceptibility of *Mycobacterium avium, Mycobacterium intracellulare*, and *Mycobacterium scrofulaceum*. Appl. Environ. Microbiol..

[143] McCarthy C. (1970). *Spontaneous* and induced mutation in *Mycobacterium avium*. Infect. Immun..

[144] Van Ingen J., Boeree M.J., van Soolingen D., Mouton J.W. (2012). Resistance mechanisms and drug susceptibility testing of nontuberculous mycobacteria. Drug Resist. Updat..

[145] Nie W.J., Xie Z.Y., Gao S., Teng T.L., Zhou W.Q., Shang Y.Y., Jing W., Shi W.H., Wang Q.F., Huang X.R. (2020). Efficacy of moxifloxacin against *Mycobacterium abscessus* in zebrafish model in vivo. Biomed. Environ. Sci..

[146] Chan E.D., Bai X. (2016). Animal models of non-tuberculous mycobacterial infections. Mycobact. Dis..

[147] Bernut A., Herrmann J.-L., Ordway D., Kremer L. (2017). The diverse cellular and animal models to decipher the physiopathological traits of *Mycobacterium abscessus* infection. Front. Cell. Infect. Microbiol..

[148] De Groote M.A., Johnson L., Podell B., Brooks E., Basaraba R., Gonzalez-Juarrero M. (2014). GM-CSF knockout mice for preclinical testing of agents with antimicrobial activity against *Mycobacterium abscessus*. J. Antimicrob. Chemother..

[149] Maggioncalda E.C., Story-Roller E., Mylius J., Illei P., Basaraba R.J., Lamichhane G. (2020). A mouse model of pulmonary *Mycobacteroides abscessus* infection. Sci. Rep..

[150] Swenson C., Zerbe C.S., Fennelly K. (2018). Host Variability in NTM disease: Implications for research needs. Front. Microbiol..

[151] Flynn J.L. (2006). Lessons from experimental *Mycobacterium tuberculosis* infections. Microbes Infect..

[152] Obregón-Henao A., Arnett K.A., Henao-Tamayo M., Massoudi L., Creissen E., Andries K., Lenaerts A.J., Ordway D.J. (2015). Susceptibility of *Mycobacterium abscessus* to antimycobacterial drugs in preclinical models. Antimicrob. Agents Chemother..

[153] Ordway D., Henao-Tamayo M., Smith E., Shanley C., Harton M., Troudt J., Bai X., Basaraba R.J., Orme I.M., Chan E.D. (2008). Animal model of *Mycobacterium abscessus* lung infection. J. Leukoc. Biol..

[154] Bernut A., Nguyen-Chi M., Halloum I., Herrmann J.-L., Lutfalla G., Kremer L. (2016). *Mycobacterium abscessus*-Induced granuloma formation is strictly dependent on TNF signaling and neutrophil trafficking. PLOS Pathog..

[155] Bolz M., Ruf M.T. (2019). Buruli ulcer in animals and experimental infection models. Buruli Ulcer: Mycobacterium Ulcerans Disease.

[156] George K.M., Pascopella L., Welty D.M., Small P.L.C. (2000). A *Mycobacterium ulcerans* toxin, mycolactone, causes apoptosis in guinea pig ulcers and tissue culture cells. Infect. Immun..

[157] George K.M., Chatterjee D., Gunawardana G., Welty D., Hayman J., Lee R., Small P.L.C. (1999). Mycolactone: A polyketide toxin from *Mycobacterium ulcerans* required for virulence. Science.

[158] Krieg R.E., Hockmeyer W.T., Connor D.H. (1974). Toxin of *Mycobacterium ulcerans*. Production and effects in guinea pig skin. Arch. Dermatol..

[159] Meijer A.H., Spaink H.P. (2011). Host-pathogen interactions made transparent with the zebrafish model. Curr. Drug Targets.

[160] Broekhuizen C.A.N., Schultz M.J., van der Wal A.C., Boszhard L., de Boer L., Vandenbroucke-Grauls C.M.J.E., Zaat S.A.J. (2008). Tissue around catheters is a niche for bacteria associated with medical device infection. Crit. Care Med..

[161] Boelens J.J., Dankert J., Murk J.L., Weening J.J., van der Poll T., Dingemans K.P., Koole L., Laman J.D., Zaat S.A.J. (2000). Biomaterial-associated persistence of *Staphylococcus epidermidis* in pericatheter macrophages. J. Infect. Dis..

[162] Busscher H.J., van der Mei H.C., Subbiahdoss G., Jutte P.C., van den Dungen J.J.A.M., Zaat S.A.J., Schultz M.J., Grainger D.W. (2012). Biomaterial-associated infection: Locating the finish line in the race for the surface. Sci. Transl. Med..

[163] Veneman W.J., Marín-Juez R., de Sonneville J., Ordas A., Jong-Raadsen S., Meijer A.H., Spaink H.P. (2014). Establishment and optimization of a high throughput setup to study *Staphylococcus epidermidis* and *Mycobacterium marinum* infection as a model for drug discovery. J. Vis. Exp..

[164] Davis J.M., Clay H., Lewis J.L., Ghori N., Herbomel P., Ramakrishnan L. (2002). Real-Time visualization of mycobacterium-macrophage interactions leading to initiation of granuloma formation in zebrafish embryos. Immunity.

[165] Johansen M.D., Kremer L. (2020). Large extracellular cord formation in a zebrafish model of *Mycobacterium kansasii* infection. J. Infect. Dis..

[166] Dupont C., Viljoen A., Thomas S., Roquet-Banères F., Herrmann J.-L., Pethe K., Kremer L. (2017). Bedaquiline inhibits the ATP synthase in *Mycobacterium abscessus* and is effective in infected zebrafish. Antimicrob. Agents Chemother..

[167] Bernut A., Le Moigne V., Lesne T., Lutfalla G., Herrmann J.-L., Kremer L. (2014). In vivo assessment of drug efficacy against *Mycobacterium abscessus* using the embryonic zebrafish test system. Antimicrob. Agents Chemother..

[168] Winthrop K., Rivera A., Engelmann F., Rose S., Lewis A., Ku J., Bermudez L., Messaoudi I. (2016). A rhesus macaque model of pulmonary nontuberculous mycobacterial disease. Am. J. Respir. Cell Mol. Biol..

[169] Peters J., Caceres D.M., Mangat M., Griffith D. (2019). Non-human primate model of Non-TB Mycobacteria (NTM) pulmonary disease. Chest.

[170] Van de Berg J.L., Williams-Blangero S. (1997). Advantages and limitations of nonhuman primates as animal models in genetic research on complex diseases. J. Med. Primatol..

[171] Vallender E.J., Miller G.M. (2013). Nonhuman primate models in the genomic era: A paradigm shift. ILAR J..

[172] Izumizono Y., Arevalo S., Koseki Y., Kuroki M., Aoki S. (2011). Identification of novel potential antibiotics for tuberculosis by in silico structure-based drug screening. Eur. J. Med. Chem..

[173] Gozalbes R., Brun-Pascaud M., Garciía-Domenech R., Gaálvez J., Girard P.-M., Doucet J.-P., Derouin F. (2000). Prediction of quinolone activity against *Mycobacterium avium* by molecular topology and virtual computational screening. Antimicrob. Agents Chemother..

[174] Garcia-Garcia A. (2003). New agents active against *Mycobacterium avium* complex selected by molecular topology: A virtual screening method. J. Antimicrob. Chemother..

[175] Schulthess P., van Wijk R.C., Krekels E.H.J., Yates J.W.T., Spaink H.P., van der Graaf P.H. (2018). Outside-in systems pharmacology combines innovative computational methods with high-throughput whole vertebrate studies. CPT Pharmacomet. Syst. Pharmacol..

[176] Pires D.E.V., Ascher D.B. (2020). mycoCSM: Using graph-based signatures to identify safe potent hits against mycobacteria. J. Chem. Inf. Model..

[177] Vente D., Arandjelović O., Baron V.O., Dombay E., Gillespie S.H. (2020). Using machine learning for automatic estimation of M. smegmatis cell count from fluorescence microscopy images. Studies in Computational Intelligence.

[178] Lee J., Rho K., Park K.H., Kim J.-S., Shin S., Kim T.S., Kim S. (2019). Utilizing Negative Markers for Identifying Mycobacteria Species based on Mass Spectrometry with Machine Learning Methods. Proceedings of the 2019 IEEE International Conference on Bioinformatics and Biomedicine (BIBM).

[179] Rastogi N., Frehel C., Ryter A., Ohayon H., Lesourd M., David H.L. (1981). Multiple drug resistance in *Mycobacterium avium*: Is the wall architecture responsible for exclusion of antimicrobial agents?. Antimicrob. Agents Chemother..

[180] Haemers A., Leysen D.C., Bollaert W., Zhang M.Q., Pattyn S.R. (1990). Influence of N substitution on antimycobacterial activity of ciprofloxacin. Antimicrob. Agents Chemother..

[181] Franzblau S.G., White K.E. (1990). Comparative in vitro activities of 20 fluoroquinolones against *Mycobacterium leprae*. Antimicrob. Agents Chemother..

[182] Chopra S., Matsuyama K., Hutson C., Madrid P. (2011). Identification of antimicrobial activity among FDA-approved drugs for combating *Mycobacterium abscessus* and *Mycobacterium chelonae*. J. Antimicrob. Chemother..

[183] Jeong J., Kim G., Moon C., Kim H.J., Kim T.H., Jang J. (2018). Pathogen box screening for hit identification against *Mycobacterium abscessus*. PLoS ONE.

[184] Low J.L., Wu M.-L., Aziz D.B., Laleu B., Dick T. (2017). Screening of TB actives for activity against nontuberculous mycobacteria delivers high hit rates. Front. Microbiol..

[185] Cowman S., Burns K., Benson S., Wilson R., Loebinger M.R. (2016). The antimicrobial susceptibility of non-tuberculous mycobacteria. J. Infect..

[186] McGuffin S., Mullen S., Early J., Parish T. (2019). 1341. Development of a series of high-throughput screens to identify leads for nontuberculous mycobacteria drug design. Open Forum Infect. Dis..

[187] Zimmer B.L., DeYoung D.R., Roberts G.D. (1982). In vitro synergistic activity of ethambutol, isoniazid, kanamycin, rifampin, and streptomycin against *Mycobacterium avium-intracellulare* complex. Antimicrob. Agents Chemother..

[188] Mukherjee D., Wu M.-L., Teo J.W.P., Dick T. (2017). Vancomycin and clarithromycin show synergy against *Mycobacterium abscessus* in vitro. Antimicrob. Agents Chemother..

[189] Machado D., Cannalire R., Santos Costa S., Manfroni G., Tabarrini O., Cecchetti V., Couto I., Viveiros M., Sabatini S. (2016). Boosting Effect of 2-phenylquinoline efflux inhibitors in combination with macrolides against *Mycobacterium smegmatis* and *Mycobacterium avium*. ACS Infect. Dis..

[190] Felicetti T., Machado D., Cannalire R., Astolfi A., Massari S., Tabarrini O., Manfroni G., Barreca M.L., Cecchetti V., Viveiros M. (2019). Modifications on C6 and C7 positions of 3-phenylquinolone efflux pump inhibitors led to potent and safe antimycobacterial treatment adjuvants. ACS Infect. Dis..

[191] Blondiaux N., Moune M., Desroses M., Frita R., Flipo M., Mathys V., Soetaert K., Kiass M., Delorme V., Djaout K. (2017). Reversion of antibiotic resistance in *Mycobacterium tuberculosis* by spiroisoxazoline SMARt-420. Science.

[192] Zheng W., Sun W., Simeonov A. (2018). Drug repurposing screens and synergistic drug-combinations for infectious diseases. Br. J. Pharmacol..

[193] Worthington R.J., Melander C. (2013). Combination approaches to combat multidrug-resistant bacteria. Trends Biotechnol..

[194] Story-Roller E., Maggioncalda E.C., Lamichhane G. (2019). Select β-lactam combinations exhibit synergy against *Mycobacterium abscessus* in vitro. Antimicrob. Agents Chemother..

[195] Hetényi C., van der Spoel D. (2006). Blind docking of drug-sized compounds to proteins with up to a thousand residues. FEBS Lett..

[196] Johnson E.O., Office E., Kawate T., Orzechowski M., Hung D.T. (2019). Discovery of a novel synergistic antimycobacterial combination targeting EfpA using large-scale chemical-genetics. bioRxiv.

[197] Wallis R.S., Hafner R. (2015). Advancing host-directed therapy for tuberculosis. Nat. Rev. Immunol..

[198] Tobin D.M. (2015). Host-Directed therapies for tuberculosis. Cold Spring Harb. Perspect. Med..

[199] Schwegmann A., Brombacher F. (2008). Host-Directed drug targeting of factors hijacked by pathogens. Sci. Signal..

[200] Hawn T.R., Shah J.A., Kalman D. (2015). New tricks for old dogs: Countering antibiotic resistance in tuberculosis with host-directed therapeutics. Immunol. Rev..

[201] Cai S., Sato K., Shimizu T., Yamabe S., Hiraki M., Sano C., Tomioka H. (2006). Antimicrobial activity of picolinic acid against extracellular and intracellular *Mycobacterium avium* complex and its combined activity with clarithromycin, rifampicin and fluoroquinolones. J. Antimicrob. Chemother..

[202] Gomes M.S., Dom G., Pedrosa J., Boelaert J.R., Appelberg R. (1999). Effects of iron deprivation on *Mycobacterium avium* growth. Tuber. Lung Dis..

[203] Pais T.F., Appelberg R. (2000). Macrophage control of mycobacterial growth induced by picolinic acid is dependent on host cell apoptosis. J. Immunol..

[204] Tanigawa K., Suzuki K., Nakamura K., Akama T., Kawashima A., Wu H., Hayashi M., Takahashi S.-I., Ikuyama S., Ito T. (2008). Expression of adipose differentiation-related protein (ADRP) and perilipin in macrophages infected with *Mycobacterium leprae*. FEMS Microbiol. Lett..

[205] Degang Y., Akama T., Hara T., Tanigawa K., Ishido Y., Gidoh M., Makino M., Ishii N., Suzuki K. (2012). Clofazimine modulates the expression of lipid metabolism proteins in *Mycobacterium leprae*-infected macrophages. PLoS Negl. Trop. Dis..

[206] Tang J., Chen Q., Guo J., Yang L., Tao Y., Li L., Miao H., Feng H., Chen Z., Zhu G. (2016). Minocycline attenuates neonatal germinal-matrix-hemorrhage-induced neuroinflammation and brain edema by activating cannabinoid receptor 2. Mol. Neurobiol..

[207] Montes-Worboys A., Brown S., Regev D., Bellew B.F., Mohammed K.A., Faruqi I., Sharma P., Moudgil B., Antony V.B. (2010). Targeted delivery of amikacin into granuloma. Am. J. Respir. Crit. Care Med..

[208] Rao M., Ippolito G., Mfinanga S., Ntoumi F., Yeboah-Manu D., Vilaplana C., Zumla A., Maeurer M. (2019). Improving treatment outcomes for MDR-TB—Novel host-directed therapies and personalised medicine of the future. Int. J. Infect. Dis..

